# Therapeutic Effects of Fenofibrate on Diabetic Peripheral Neuropathy by Improving Endothelial and Neural Survival in *db/db* Mice

**DOI:** 10.1371/journal.pone.0083204

**Published:** 2014-01-02

**Authors:** Ye Rim Cho, Ji Hee Lim, Min Young Kim, Tae Woo Kim, Bo Young Hong, Yong-Soo Kim, Yoon Sik Chang, Hye Won Kim, Cheol Whee Park

**Affiliations:** 1 Department of Internal Medicine, Seoul St. Mary's Hospital, College of Medicine, The Catholic University of Korea, Seoul, Korea; 2 Rehabilitation Medicine, Seoul St. Mary's Hospital, College of Medicine, The Catholic University of Korea, Seoul, Korea; Children's Hospital Boston, United States of America

## Abstract

Neural vascular insufficiency plays an important role in diabetic peripheral neuropathy (DPN). Peroxisome proliferative-activated receptor (PPAR)α has an endothelial protective effect related to activation of PPARγ coactivator (PGC)-1α and vascular endothelial growth factor (VEGF), but its role in DPN is unknown. We investigated whether fenofibrate would improve DPN associated with endothelial survival through AMPK-PGC-1α-eNOS pathway. Fenofibrate was given to *db/db* mice in combination with anti-flt-1 hexamer and anti-flk-1 heptamer (VEGFR inhibition) for 12 weeks. The *db/db* mice displayed sensory-motor impairment, nerve fibrosis and inflammation, increased apoptotic cells, disorganized myelin with axonal shrinkage and degeneration, fewer unmyelinated fibers, and endoneural vascular rarefaction in the sciatic nerve compared to *db/m* mice. These findings were exacerbated with VEGFR inhibition in *db/db* mice. Increased apoptotic cell death and endothelial dysfunction via inactivation of the PPARα-AMPK-PGC-1α pathway and their downstream PI3K-Akt-eNOS-NO pathway were noted in *db/db* mice, human umbilical vein endothelial cells (HUVECs) and human Schwann cells (HSCs) in high-glucose media. The effects were more prominent in response to VEGFR inhibition. In contrast, fenofibrate treatment ameliorated neural and endothelial damage by activating the PPARα-AMPK-PGC-1α-eNOS pathway in *db/db* mice, HUVECs and HSCs. Fenofibrate could be a promising therapy to prevent DPN by protecting endothelial cells through VEGF-independent activation of the PPARα-AMPK-PGC-1α-eNOS-NO pathway.

## Introduction

Diabetic peripheral neuropathy (DPN) affects at least 50% of patients with diabetes, and is the leading cause of foot amputation [1], [2]. Hyperglycemia is likely the primary factor [3], which is associated with changes in endoneural metabolism, including increases in the polyol pathway, advanced glycation and protein kinase C, impairment of essential fatty acid metabolism, defective neurotropic factors, and reduced nerve blood supply [4], . Human studies have found various histopathological anomalies in the sural nerve in patients with diabetes related to reduced blood flow [4]. Stevens et al. found that nerve blood flow fell by 80% on the fourth day following the induction of diabetes in rats [8]. Early physiological disorders such as nerve conduction showing and sensory loss can be used diagnosis, which is associated with reduced oxygen tension and peripheral vascular disease, and may predict onset of degenerative changes in neurons, Schwann cells and blood vessel [4], [9], [10]. The most widely and consistently reported structural change in the nerve trunks of streptozotocin-diabetic rats is reduced axonal caliber of myelinated fibers [11], [12]. However, rodent models of DPN are frequently faulted as showing little reliable evidence of overt structural damage to myeline fiber such as the segmental demyelination, remyelination and Wallerian degeneration that characterizes human DPN [13].

Vascular endothelial growth factor (VEGF) is a critical component during the tissue growth and organ repair processes of angiogenesis and vasculogenesis and also is a survival factor for endothelial cells [14]. The two primary VEGF receptors on the vascular endothelium are VEGFR-1 (flt-1) and VEGFR-2 (flk-1). Besides its physiological actions, VEGF is vital for promoting the formation of collateral vessels after ischemic events and plays a key role in wound healing [14], [15]. Moreover, it is clear that VEGF treatment via VEGFR-2 promotes neurotropic effects in peripheral nervous system cells characterized by Schwann cell proliferation, stimulation of axonal outgrowth, and increased survival in both neurons and Schwann cells [16], [17]. While therapeutic angiogenesis with VEGF and related molecules once held great promise for the treatment of DPN, it has not been successful to date. Therefore, alternative strategies that aim to stimulate revascularization of ischemic tissue are warrented [18], [19]. Arany and colleagues recently reported that peroxisome proliferator-activated receptor-γ coactivator 1α (PGC-1α) simulates angiogenesis in ischemic tissues [19]. They demonstrated that PGC-1α up-regulates angiogenic factors other than VEGF and stimulates revascularization of ischemic tissue. PGC-1α interacts with and coactivates many members of the nuclear receptor transcription factor superfamily. In the cardiovascular system, three major PGC-1α transcription factor partners have been identified; peroxisome proliferator-activated receptor (PPAR)α, estrogen related receptor (ERR) family and nuclear respiratory factor 1 (NRF-1) [20].

Lipid-lowering therapy with a fibrate may have benefits for DPN beyond its anti-atherogenic effects in type 2 diabetes [21]. Activation of PPARα attenuates or inhibits several vascular damage mediators, including lipotoxicity, inflammation, reactive oxygen species generation, endothelial dysfunction, and thrombosis. These protective effects may be influenced by the intracellular Phosphatidylinositol 3 kinase (PI3K)-Akt-endothelial nitric oxide synthase (eNOS) signaling pathway that underlies diabetic neuropathy [22], [23], [24]. Furthermore, fenofibrate stimulates AMP-activated kinase (AMPK)-eNOS expression in HUVECs, thereby increasing nitric oxide (NO) production, inhibiting NF-κB, and suppressing cellular adhesion molecules [24], [25]. Pharmacological treatment with fibrate increases PGC-1α due to an increase in mitochondrial biogenesis [26]. Moreover, direct regulation of PGC-1α though activation of PPARα has also been suggested [27].

Therefore, we investigated whether fenofibrate had a protective role against DPN in *db/db* mice through PGC-1α activation during blockade of VEGF-VEGFR signaling in diabetic mice. A selective anti-flt1 hexamer (anti-flt-1; GNQWFI) and an anti-flk-1 heptamer (anti-flk-1; ATWLPPR) inhibit VEGF-induced endothelial cell migration and morphogenesis via VEGR-R1 inhibition [28] and VEGF-induced angiongenesis and endothelial cell proliferation via VEGR-R2 inhibition, respectively, in both in vivo and in vitro model [19], [20], [21].

## Materials and Methods

### Experimental Methods

All experiments were performed in accordance with the institutional animal care guidelines, and all procedures complied with the *Guide for the Care and Use of Laboratory Animals* (National Institutes of Health Publication No. 85-23, revised 1996). Because loss of a single VEGF allele is lethal in mice embryos due to impaired angiogenesis and blood-island formation from P11-12 [28], VEGFR-1 and VEGFR-2 blockade was induced in the mice with a combination of GNQWFI (50 µg/kg) and ATWLPPR (50 µg/kg) peptides were subcutaneously injected into diabetic (*db/db* VEGFR12, *n* = 8) and nondiabetic (*db/m* VEGFR12, *n* = 8) mice daily for 12 weeks beginning at 8 weeks of age [29], [30], [31]. Fenofibrate (0.2%, w/w, Sigma, St. Louis, MO, USA) was given in the standard chow diet to diabetic mice (*db/db*+Feno), diabetic VEGFR-1, 2 mice (*db/db* VEGFR12+Feno, *n* = 8), non-diabetic mice (*db/m*+Feno), and non-diabetic VEGFR-1, 2 mice (*db/m* VEGFR12+Feno, *n* = 8) from 8–20 weeks of age. The control groups (*db/m, db/db*) and untreated VEGFR-1, 2 blocking groups (*db/m* VEGFR12, *db/db* VEGFR12) received normal mouse chow for 12 weeks.

After 12 weeks of fenofibrate treatment, we performed electrophysiological and behavioral tests in the following order: tactile responses to stimulation using flexible von Frey filaments and then sciatic motor nerve conduction studies (MNCS). After the tests were completed, we measured serum parameters and the sciatic nerves were collected under general anesthesia. Some sciatic nerve samples were fixed in normal buffered 4% formalin for immunohistochemistry, and the others were stored in a solution for electron microscopy.

### Ethics Statement

The study was carried out in strict accordance with the recommendations of the Guide for the Care and Use of Laboratory Animals of the National Institutes of Health. The protocol was approved by the Laboratory Animal Care Committee at the Catholic University of Korea (Permit Number; CUMC- 2012-0118).

### Assessment of Peripheral Nerve Function

#### Tactile responses and Sciatic motor nerve conduction studies (MNCS)

Tactile responses were evaluated by quantifying the 50% paw withdrawal threshold in response to stimulation with flexible von Frey filaments (Touch-Test™ Sensory Evaluators, Stoelting Co., Wooddale, IL) as described by Chaplan et al [32]. MNCS was performed on the left sciatic nerve using an electromyographic device (Synergy, Medelec Ltd., Oldwoking, England), within 15 min of isoflurane anesthesia to avoid respiratory arrest. The sciatic nerve was stimulated supra-maximally (2–3 mA, 0.1 ms) at the ipsilateral sciatic notch and motor response was obtained from the fifth inter-osseous muscle of the hindpaw. Compound muscle action potentials were measured from the stimulus to the onset of the negative M-wave deflection.

### Blood Glucose, HbA1c and Lipid Profiles

After 12 weeks of treatment with fenofibrate, blood glucose was measured using an Accucheck meter (Roche Diagnostics, St. Louis, MO). Hemoglobin A1c (HbA1c) was determined on red cell lysates using high-performance liquid chromatography (BioRab, Hercules, CA). Total cholesterol (TC), high-density lipid cholesterol (HDL-C) and triacylglycerol (TG) concentrations were measured with an auto-analyzer (Hitachi 917, Tokyo, Japan) using commercial kits (Wako, Osaka, Japan).

### Light- and Electron-microscopic Analysis

#### Nerve morphology

Sciatic nerve samples were collected and fixed in 4% paraformaldehyde. Trichrome-stained nerves were used to examine the effect of VEGFR inhibition on nerve fibrosis. Ten consecutive nerve cross-sections were photographed using a digital camera (Olympus DP11; Olympus America, Melville, NY) by an examiner who was blinded to the tissue source. Each nerve section was sampled in a serpentine pattern such that the entire nerve section was analyzed with no overlapping fields. The number of endoneurial blood vessels was counted. Artifacts and non-ideal axonal cross sections (tangential or paranodal profiles) were excluded.

#### Immunohistochemical techniques for transforming growth factor-β1 (TGF-β1), platelet-endothelial cell adhesion molecule-1 (PECAM-1), F4/80, 8-hydroxy-deosyguanosine (8-OH-dG), PPARα and the terminal deoxynucleotidyl transferase dUTP nickend-labeling (TUNEL) assay

We performed immunohistochemistry for TGF-β1, PECAM-1, 8-OH-dG, PPARα and the TUNEL assay. Four-µm-thick sections were incubated overnight in a humidified chamber at 4°C with anti-TGF-β1, a profibrotic growth factor (1∶100; R&D Systems, Minneapolis, MN); anti-PECAM-1, a mouse endothelial cell marker (1∶100; Abcam, Cambridge, UK); anti-F4/80, an inflammatory cell marker (1∶100; Santa Cruz Biotechnology, CA); the *in situ* TUNEL assay, an apoptotic marker (Chemicon-Millipore, Billerica, MA); 8-OH-dG, an oxidative DNA damage marker (1∶100; COSMO BIO, Tokyo, Japan); and PPARα (1∶200; Abcam, Cambridge, UK).

#### Immunofluorescence staining for DAPI, TUNEL and PECAM-1

For immunofluorescence double staining, apoptosis was detected by ApopTag Fluorescein In Situ Apoptosis Detection Kit (S7110; Chemicon International, Temecula, CA). The ApopTag Fluorescence In Situ Apoptosis Detection Kit detects apoptotic cells in situ by the indirect TdT-mediated dUTP-biotin nick end labeling (TUNEL) method, using an anti-digoxigenin antibody that is conjugated to a fluorescence reporter molecule. Sections were incubated overnight with anti-PECAM-1 (1∶50; Abcam, Cambridge, UK) and a Texas red-labeled secondary antibody and counterstained with the 4,6-diamidino2-phenylindole (DAPI).

#### Electron microscopy

For transmission electron microscopy (TEM), sciatic nerves specimens were fixed in 4% paraformaldehyde and 2.5% glutaraldehyde in 0.1 M phosphate buffer overnight at 4°C. After washing in 0.1 M phosphate buffer, the specimens were post-fixed with 1% osmium tetroxide in the same buffer for 1 hr. The specimens were then dehydrated using a series of graded ethanol, exchanged through acetone, and embedded in Epon 812. Ultrathin sections (70∼80 nm) were obtained by ultramicrotome (Leica Ultracut UCT, Leica, Germany) and were double stained with uranyl acetate and lead citrate and examined in transmission electron microscope (JEM 1010, Tokyo, Japan) at 60 kV. We measured areas of unmyelinated fiber, axonal diameters and number of degenerative fiber using NIH Image J.

### Western Blot Analysis

Western blot analysis was performed using the following antibodies: TGF-β1 (1∶500; R&D Systems, Minneapolis, MN), platelet endothelial cell adhesion molecule (PECAM-1, 1∶500; Abcam, Cambridge, UK), PPARα (1∶1000; Abcam, Cambridge, UK), PGC-1α (1∶2000; Novus Biologicals, Littleton, CO), total-AMPK (1∶1000; Cell Signaling Technology, Danvers, MA), phospho-Thr^172^ AMPK (1∶1000; Cell Signaling Technology, Danvers, MA), HIF-1α(1: 500; Abcam, Cambridge, UK), and β-actin (1∶6000; Sigma-Aldrich, St Louis, MO). Protein for HIF-1α from nuclear fractions of the nerve was isolated using a nuclear extraction kit (Cayman Chemical, Ann Arbor, MI) following the manufacturer's protocol.

### Western Blot Analysis, TUNEL Assay, and NOx Levels in the HUVECs and Human Schwann Cells (HSCs) Culture

Human umbilical vein endothelial cells (HUVECs, Lonza, Postsmouth, NH), which are commonly used for VEGF-dependent signaling studies, were cultured endothelial growth medium (EGM-2, Lonza, Postsmouth, NH) at 37°C in a humidified, 5% CO2/95% air atmosphere. Human Schwann cells (HSCs, ScienCell Research Laboratories, San Diego, CA) were also cultured Schwann Cell Medium (SCM, ScienCell Research Laboratories, San Diego, CA) at 37°C in a humidified, 5% CO2/95% air atmosphere. Passages 4–8 were used in all experiments. Apoptosis was quantified using the *in situ* cell death detection kit by TUNEL assay (Chemicon International, Temecula, CA). After treatment with different concentrations of D-glucose in the media (5 mmol/L D-glucose; low-glucose, 30 mmol/L D-glucose; high-glucose, and D-glucose [5 mmol/L]+D-mannitol [25 mmol/L]; osmotic control) including 10^−8^ mol/L VEGFR12 with or without 50 µmol/L fenofibrate for 48 hr, the number of TUNEL-positive cells was counted in 10 randomly chosen fields at a magnification of 400×. We also performed Western blot analysis using 50 µmol/L fenofibrate for 48 hr with the following antibodies: PPARα (1∶1000; Abcam, Cambridge, UK), PGC-1α (1∶2000; Novus Biologicals, Littleton, CO), total-AMPK (1∶1000; Cell Signaling Technology, Danvers, MA), phospho-Thr^172^ AMPK (1∶1000; Cell Signaling Technology, Danvers, MA), PI3K (1∶1500; BD Biosciences, Franklin Lakes, NJ), total Akt, phospho-Ser^473^ Akt, total endothelial nitric oxide synthase (eNOS), and phospho-Ser^1177^ eNOS (1∶1000; Cell Signaling Technology, Danvers, MA), and β-actin (Sigma-Aldrich, St Louis, MO). We also measured the concentration of NOx to quantify NO production in cell-culture media. We also performed similar experiment using Schwann cells.

### Data Analysis

SPSS version 11.5 (SPSS. Inc., Chicago, IL) was used to conduct the statistical analysis. Group differences were evaluated using an analysis of variance (ANOVA) with Bonferonni's correction. Non-normally distributed data were analyzed by the Mann-Whitney *U*-test. The results are expressed as mean ± SD. A *P*<0.05 was considered significant.

## Results

### Body Weights, Glucose Levels, HbA1c Concentrations, and Lipid Profiles

At the study's conclusion, the average body weight of *db/db* mice was greater than that of *db/m* mice in both the VEGFR-inhibited and control groups (Table 1; *P*<0.001). No changes in body weight were noted in either *db/m* or *db/db* mice (with or without VEGFR inhibition) following the 12-week treatment with fenofibrate. Fasting blood sugar levels, HbA1c concentrations, and serum TG and TC levels were significantly higher in *db/db* mice than in *db/m* mice in both the VEGFR-inhibited and control groups (Table 1; *P*<0.001). Fenofibrate treatment resulted in a moderate yet significant decrease in both fasting blood glucose levels and HbA1c concentrations when compared with both non-treated diabetic *db/db* and *db/db* VEGFR12 mice (*P*<0.05). However, fenofibrate treatment did not affect serum TG levels of either *db/db* or *db/m* mice. In contrast, serum TC levels increased in both *db/db* and *db/db* VEGFR12 mice (*P*<0.05). Increased HDL-C levels were also observed in both *db/db*+Feno and *db/db* VEGFR12+Feno mice following fenofibrate treatment.

**Table 1:** Body weight and biochemical characteristics of *db/m* and *db/db* mice under VEGFR inhibition treated with fenofibrate.

Parameters	*db/m*				*db/db*			
	cont	Feno	VEGFR12	VEGFR12+Feno	cont	Feno	VEGFR12	VEGFR12+Feno
Body weight (g)	31.6±1.6	28.9±2.3	31.4±2.2	29.3±0.9	49.5±3.3**	46.3±3.5**	44.3±4.6**	51.8±3.1**
Glucose (mmol/l)	9.3±0.6	8.5±1.1	10.2±0.9	8.4±0.7	29.1±3.6**	21.7±4.3** §	27.5±4.1**	20.1±7.4** §
HbA1c (%)	5.2±0.2	5.1±0.3	4.8±0.4	5.2±0.2	11.3±1.5**	9.75±2.0** §	11.1±1.9**	9.42±1.7** §
TC (mmol/l)	27.9±2.9	25.9±2.7	28.6±2.4	25.4±2.2	44.1±4.7*	55.6±4.7** §	39.6±3.9*	63.1±4.9** §
HDL-C (mmol/l)	1.8±0.2	1.7±0.3	1.8±0.3	1.6±0.2	3.7±0.3*	4.8±0.3** §	3.4±0.2*	4.9±0.2** §
TG (mmol/l)	1.05±0.29	1.28±0.23	1.22±0.15	1.34±0.28	1.72±0.17*	1.59±0.21*	1.96±0.14*	1.74±0.22*

Abbreviations: Feno; fenofibrate, TC; total cholesterol, HDL-C; high density lipoprotein-cholesterol, TG; triacylglycerol, VEGFR12; VEGFR-1 and –2 inhibition.

*P*<0.01,

*P*<0.001 compared with *db/m*, *db/m*+VEGFR12, and *db/m*+VEGFR12+Feno groups.

*P*<0.05 compared with *db/db* and *db/db*+VEGFR12 groups.

### Assessment of Peripheral Nerve Function

#### Tactile responses and sciatic motor nerve conduction

**Figure 1:**
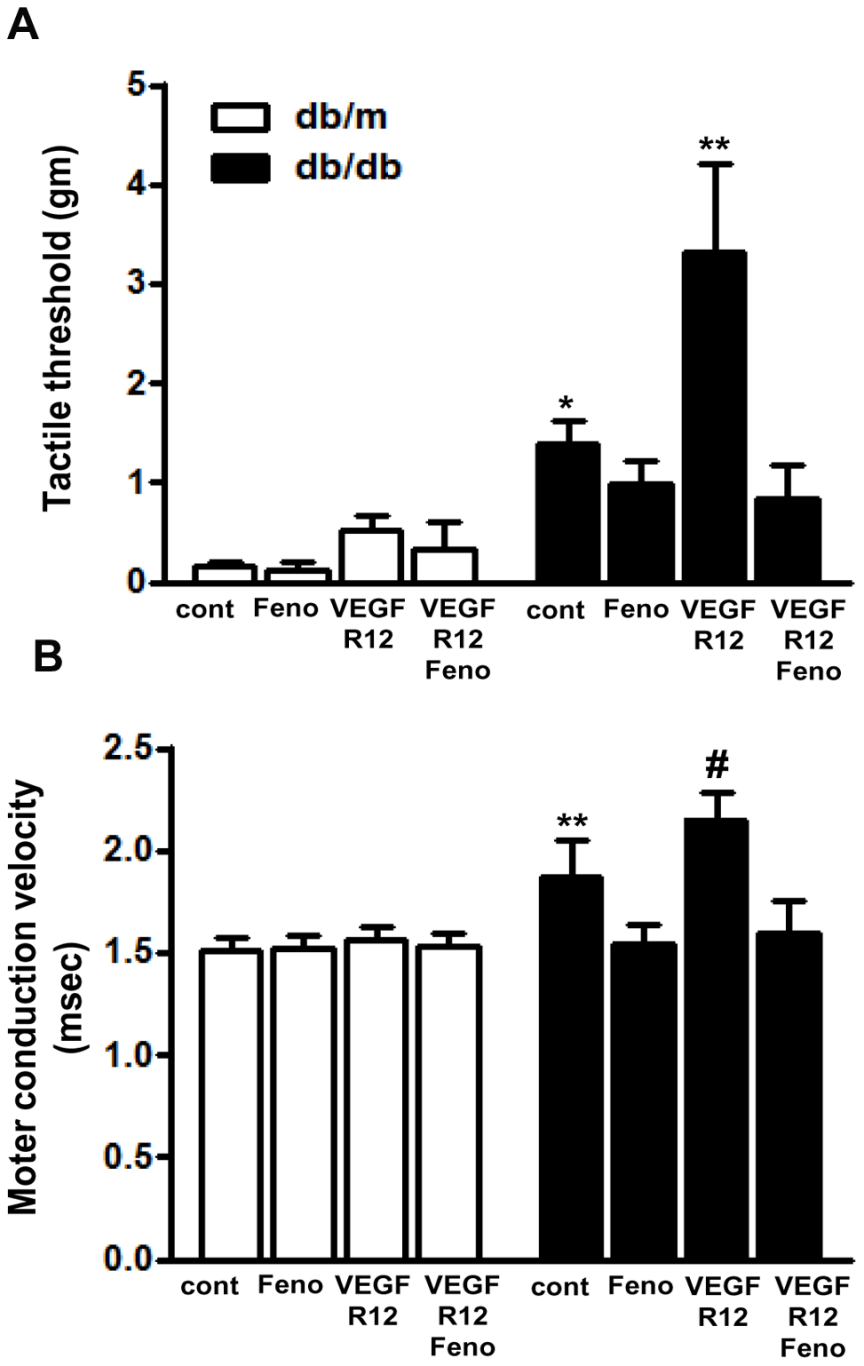
Fenofibrate improves sensory and motor functions of the sciatic nerve in *db/db* mice with VEGF inhibition. Tactile threshold (A) and motor conduction latency of the sciatic nerves (B) in *db/m* and *db/db* mice after 12 weeks of vascular endothelial growth factor receptor (VEGFR) inhibition plus fenofibrate or VEGFR inhibition alone. *P<0.05, **P<0.01 and ^#^ *P*<0.001 versus *db/m* controls, *db/m*+Feno, *db/m* VEGFR12, *db/m* VEGFR12+Feno, *db/db*+Feno, and *db/db* VEGFR12+Feno mice.

Tactile response thresholds were increased in *db/db* mice compared to control *db/m* mice. Furthermore, the average tactile threshold response of *db/db* VEGFR12 mice exhibited a significant 2.6-fold increase compared to that of *db/db* mice. Importantly, fenofibrate treatment rescued tactile response thresholds in both *db/db*+Feno and *db/db* VEGFR12+Feno mice to levels comparable to that of *db/m* mice (Figure 1).

Sciatic motor latency was significantly delayed in *db/db* mice compared to *db/m* mice (Figure 1, 1.87±0.18 vs. 1.51±0.06 msec, *P*<0.01); furthermore, the sciatic motor latency delay in *db/db* VEGFR12 mice (2.15±0.18 msec) was significantly greater than that in *db/db* mice (*P*<0.05). Consistent with their improved tactile responses, fenofibrate treatment restored sciatic motor latency in *db/db*+Feno and *db/db* VEGFR12+Feno mice to levels similar to that of non-diabetic *db/m* mice (1.59±0.16 vs. 1.51±0.06 msec). These findings demonstrate that VEGFR inhibition significantly impairs sciatic motor nerve conduction in *db/db* mice, and that this impairment can be reversed by fenofibrate treatment.

### Assessment of Nerve Pathology

#### Development of nerve fibrosis and expression of TGF-β1, PECAM-1, and HIF-1α

**Figure 2:**
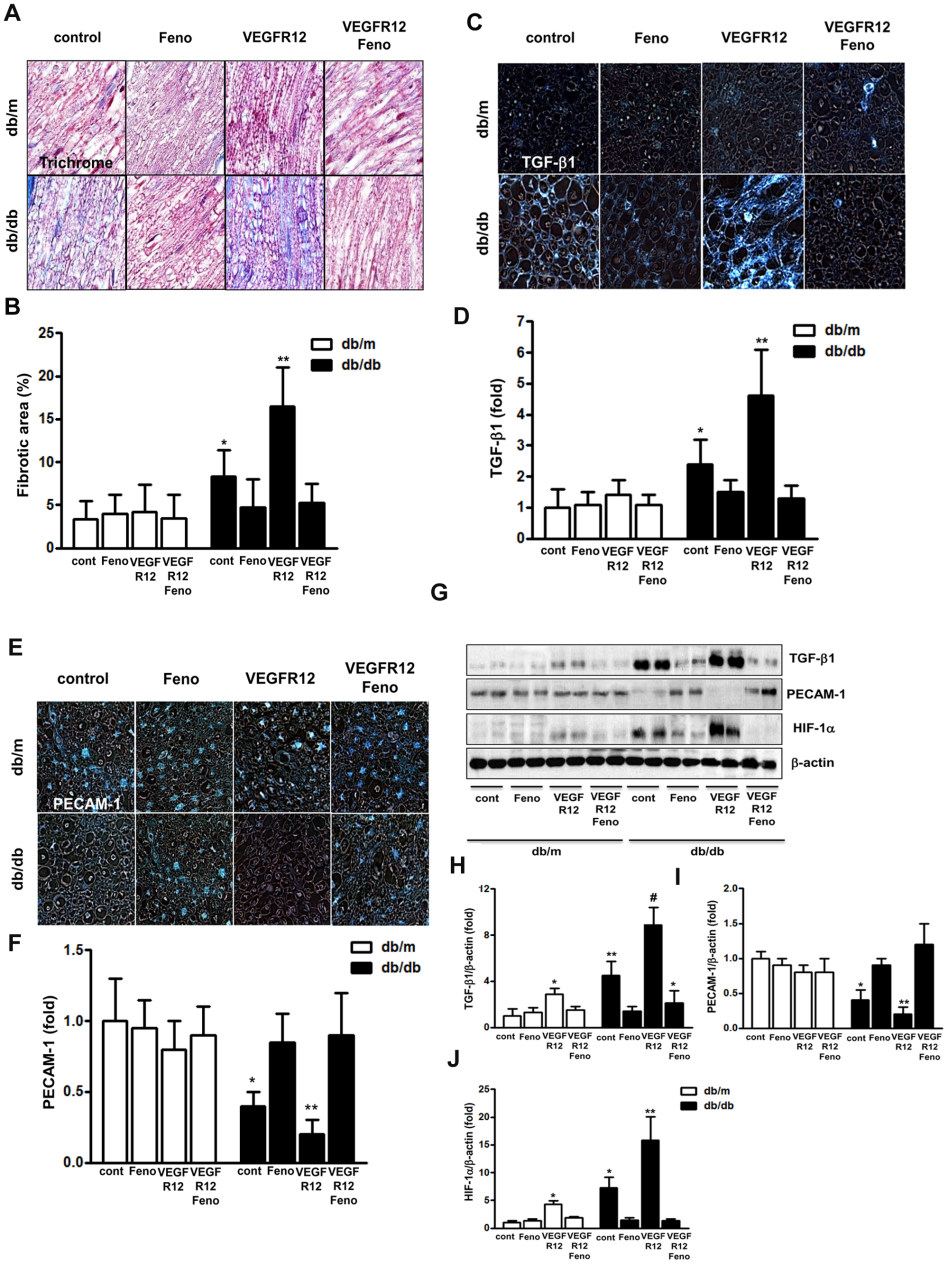
Fenofibrate attenuates fibrosis and ischemia of the sciatic nerve in *db/db* mice with VEGF inhibition. Nerve fibrosis, pro-fibrotic transforming growth factor (TGF)-β and platelet endothelial cell adhesion molecule (PECAM)-1 expression in the sciatic nerves of *db/m* and *db/db* mice after 12 weeks of VEGFR inhibition plus fenofibrate or VEGFR inhibition alone. Representative Masson's trichrome staining and quantitative analysis of the fibrotic area (1000×) (A). Representative immunohistochemical staining for TGF-β1 and PECAM-1 (mouse endothelial cell maker) and quantitative analyses of the results are shown (C to F). Representative Western blot analysis of TGF-β1, PECAM-1 and hypoxia-inducible factor (HIF)-1α (a marker for hypoxia) and quantitative analyses of the results are shown (G to J). *P<0.05 and **P<0.01 versus *db/m* controls, *db/m*+Feno, *db/m* VEGFR12, *db/m* VEGFR12+Feno, *db/db*+Feno and *db/db* VEGFR12+Feno mice (A to D). *P<0.05, **P<0.01 and ^#^ *P*<0.001 versus *db/m* controls.

In *db/db* mice, the sciatic nerve showed significantly more fibrosis in the trichrome-stained area than in either *db/m* or *db/m* VEGFR12 mice (Figure 2). Furthermore, immunohistochemical staining and Western blot analysis revealed a higher level of TGF-β1 expression in the sciatic nerve of *db/db* mice than in that of *db/m* and *db/m* VEGFR12 mice (Figure 2; *P*<0.05). TGF-β1 expression levels were also higher in *db/db* VEGFR12 mice than in *db/db* mice (*P*<0.01). Thus, a 12-week fenofibrate treatment course completely protected *db/db* and *db/db* VEGFR12 mice from increased sciatic nerve fibrosis and upregulation of TGF-β1 expression (*P*<0.01). Immunohistochemical staining and Western blot analysis also revealed a marked decrease in PECAM-1 expression and an increase in HIF-1α expression in the sciatic nerve of control *db/db* mice compared with that of non-diabetic *db/m* mice (Figure 2). Furthermore, PECAM-1 was barely detected in the sciatic nerve of *db/db*+VEGFR12 mice (*P*<0.05), and HIF-1α expression was increased even more in *db/db*+VEGFR12 mice than in *db/db* mice. In contrast, PECAM-1 and HIF-1α expression levels in *db/db*+Feno and *db/db* VEGFR12+Feno mice were restored to those of *db/m* mice. These effects were only observed upon fenofibrate treatment, and were not observed in untreated *db/m* mice.

#### F4/80-positive cells, 8-OH-dG expression, TUNEL- and PECAM-1-positive cells and PECAM-1 and TUNEL-double positive cells in the sciatic nerve

**Figure 3:**
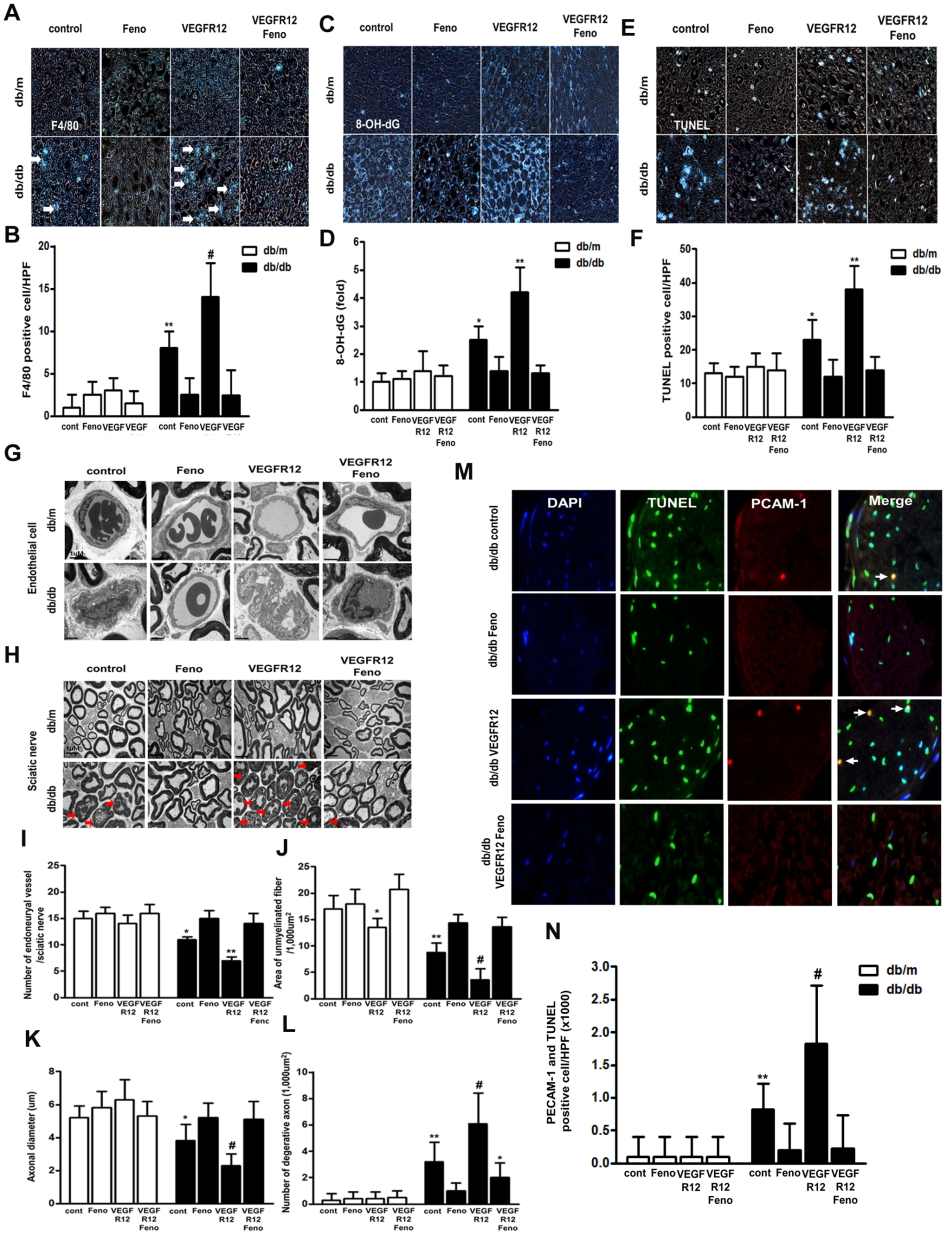
Fenofibrate decreases inflammatory cell infiltration, oxidative stress and apoptosis of the sciatic nerve in *db/db* mice with VEGF inhibition. Representative immunohistochemical stain for F4/80-, 8-OH-dG- and TUNEL-positive cells and quantitative analyses of the results are shown (A to F). *P<0.05, **P<0.01 and ^#^ *P*<0.001 versus *db/m* controls. Representative electron microscopic images of the sciatic nerve endoneural endothelial cells (15,000×) and nerve bundles (5,000×) (G and H). Disorganized, vacuolized and thickened endothelial cells were observed in *db/db* controls and *db/db* VEGFR12 mice. These characteristics decreased after 12-week fenofibrate treatment in the *db/db*+Feno and *db/db* VEGR12+Feno mice. A marked decrease in the number of unmyelinated nerve bundles (red arrowheads) and prominent axonal degeneration (red arrows) were observed in the *db/db* controls and *db/db* VEGFR12 mice. These deficits were also improved by the 12-week fenofibrate treatment in the *db/db*+Feno and *db/db* VEGR12+Feno mice. Quantitative analyses of number of endoneurial blood vessel, area of unmyelinated fiber, axonal diameter and number of degenerated axon are shown (I to L). *P<0.05 versus other groups, *P<0.05, **P<0.01, and ^#^ *P*<0.001 versus *db/m* controls. Representative immunofluorescent stains for PECAM-1 and TUNEL-double positive cells in *db/db* mice groups and quantitative analyses of the results are shown (M and N). An increase in the number of PECAM-1 and TUNEL-double positive cells (white arrows) was observed in the *db/db* controls and *db/db* VEGFR12 mice. **P<0.01 and ^#^ *P*<0.001 versus *db/m* controls.

Inflammatory cell infiltration, as defined by the presence of F4/80-positive cells, was more severe in *db/db* mice compared with non-diabetic *db/m* mice. Inflammatory cell infiltration was also more severe in *db/db* VEGFR12 mice than in *db/db* mice. Moreover, both *db/db* and *db/db* VEGFR12 mice showed higher levels of 8-OH-dG compared with *db/m* mice, reflecting increased neuronal oxidative stress (Figure 3). In both fenofibrate-treated groups, *db/db*+Feno and *db/db* VEGFR12+Feno, production of 8-OH-dG decreased to levels comparable to that of *db/m* mice. However, VEGFR inhibition and fenofibrate treatment of *db/m* mice did not produce significant changes in either inflammatory cell infiltration or 8-OH-dG production. VEGFR inhibition increased the number of TUNEL-positive cells in *db/db* mice (Figure 3); however, compared with *db/db* VEGFR12 mice, fenofibrate treatment reduced the number of TUNEL-positive cells in *db/db* mice by one-third, resulting in a number comparable to that of *db/m* mice. Diabetes induced a significant decrease in the density of endoneurial blood vessels in the sciatic nerve, an effect which was even more pronounced upon VEGFR inhibition (Figure 3). In contrast, fenofibrate treatment increased the number of endoneurial blood vessels. In order to further characterize the population of TUNEL-positive cells, we stained simultaneously for both TUNEL and PECAM-1. Consistent with the TUNEL-positive cell changes observed in the previous experiments, the number of PECAM-1 and TUNEL-double positive cells was increased in *db/db* mice, and was increased to an even greater extent in *db/db* VEGFR12 mice (Figure 3). Particularly noteworthy was the finding that fenofibrate treatment of both *db/db* and *db/db* VEGFR12 mice reduced the numbers of PECAM-1 and TUNEL-double positive cells to ones comparable to those of *db/m* mice. In contrast, no difference was observed in the number of PECAM-1 and TUNEL-double positive cells in *db/m* mice upon fenofibrate treatment (Figure 3 and data not shown). These findings suggest that vascular irregularities contribute to diabetes pathogenesis, and also that vascular irregularities combined with VEGFR inhibition drive further vascular dysfunction in the sciatic nerve by increasing endothelial cell apoptosis in the context of diabetes.

#### Electron microscopy

As assessed by ultrastructural studies, the sciatic nerve of *db/db* mice exhibited a decreased total area of unmyelinated fiber and decreased axonal diameter; furthermore, the sciatic nerve of *db/db* mice showed increased axonal degeneration (Figure 3). Diabetic mice with VEGFR inhibition also showed marked reductions in unmyelinated fiber area and axonal diameter, and a similar increase in axonal degeneration compared to diabetic mice (Figure 3). Of particular interest, sciatic nerves from fenofibrate-treated *db/db* mice (with or without VEGFR inhibition) harbored ultra-micro structures, resembling sciatic nerves from *db/m* control mice, in which the numbers of endoneurial blood vessels, areas of unmyelinated fiber, axonal diameters, and degrees of axonal degeneration were comparable to those of *db/m* control mice. Moreover, endoneurial blood vessels displayed endothelial cell disorganization, with basement membrane thickening in diabetic *db/db* VEGFR12 mice (Figure 3). The morphologies of endoneurial blood vessels in *db/db*+Feno, *db/db* VEGFR12+Feno, and *db/m* mice were all comparable.

#### Sciatic nerve expression of PPARα, PGC-1α, total-AMP and phospho-AMPK(Thr^172^)

**Figure 4:**
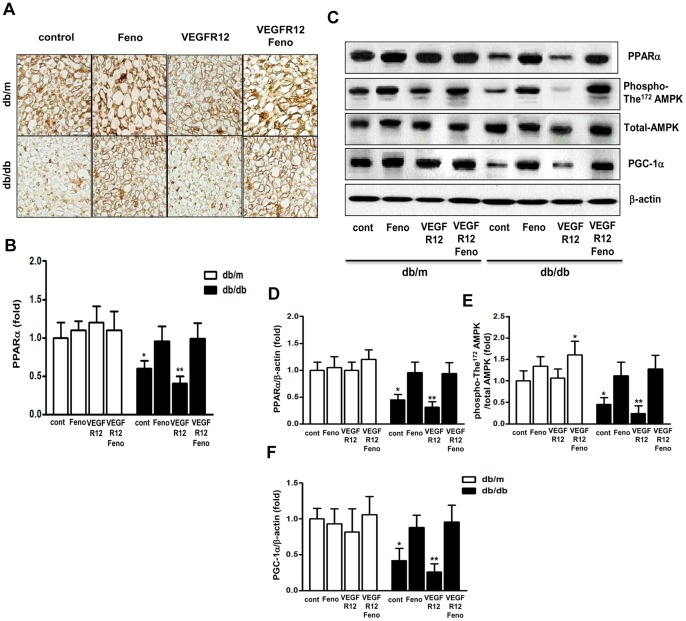
Fenofibrate upregulates the expression of PPARα-PGC-1α-phospho-The^172^ AMPK in *db/db* mice with VEGF inhibition. Peroxisome proliferative-activated receptor (PPAR)α, PPARγ coactivator (PGC)-1α, phospho-The^172^/total AMPK and β-actin expression in the sciatic nerves of *db/m* and *db/db* mice after 12 weeks VEGFR inhibition plus fenofibrate or VEGFR inhibition alone. Representative immunohistochemical staining of PPARα and quantitative analyses of the results are shown (A and B). *P<0.05 versus *db/m* controls. Western blot analysis of PPARα, PGC-1α, phospho-The^172^/total AMPK and β-actin (C, respectively) and quantitative analyses of the results are shown. *P<0.05 and **P<0.01 versus *db/m* controls.

Immunohistochemical staining and Western blot analysis were next performed, and revealed that PPARα expression levels in *db/db* mice decreased significantly, by 40% and 60% respectively, compared with *db/m* mice (Figure 4). However, VEGFR inhibition in *db/m* mice did not change PPARα expression levels compared to *db/m* mice, whereas VEGF inhibition in *db/db* mice further decreased PPARα expression levels. Significantly, fenofibrate treatment increased PPARα expression levels in *db/db*+Feno and *db/db* VEGFR12+Feno mice to those of non-diabetic *db/m* mice. Furthermore, both PGC-1α expression levels and the ratios of phospho-Thr^172^/total AMPK were lower in *db/db* mice compared to those in *db/m* mice; VEGFR inhibition further decreased these levels in *db/db* mice (Figure 4; *P*<0.05). However, the 12-week fenofibrate treatment course increased PGC-1α and phospho-AMPK(Thr^172^) expression levels in *db/db* mice to levels comparable to those of *db/m* mice.

### Effects of VEGFR Inhibition and Fenofibrate Treatment on HUVECs and HSCs

**Figure 5:**
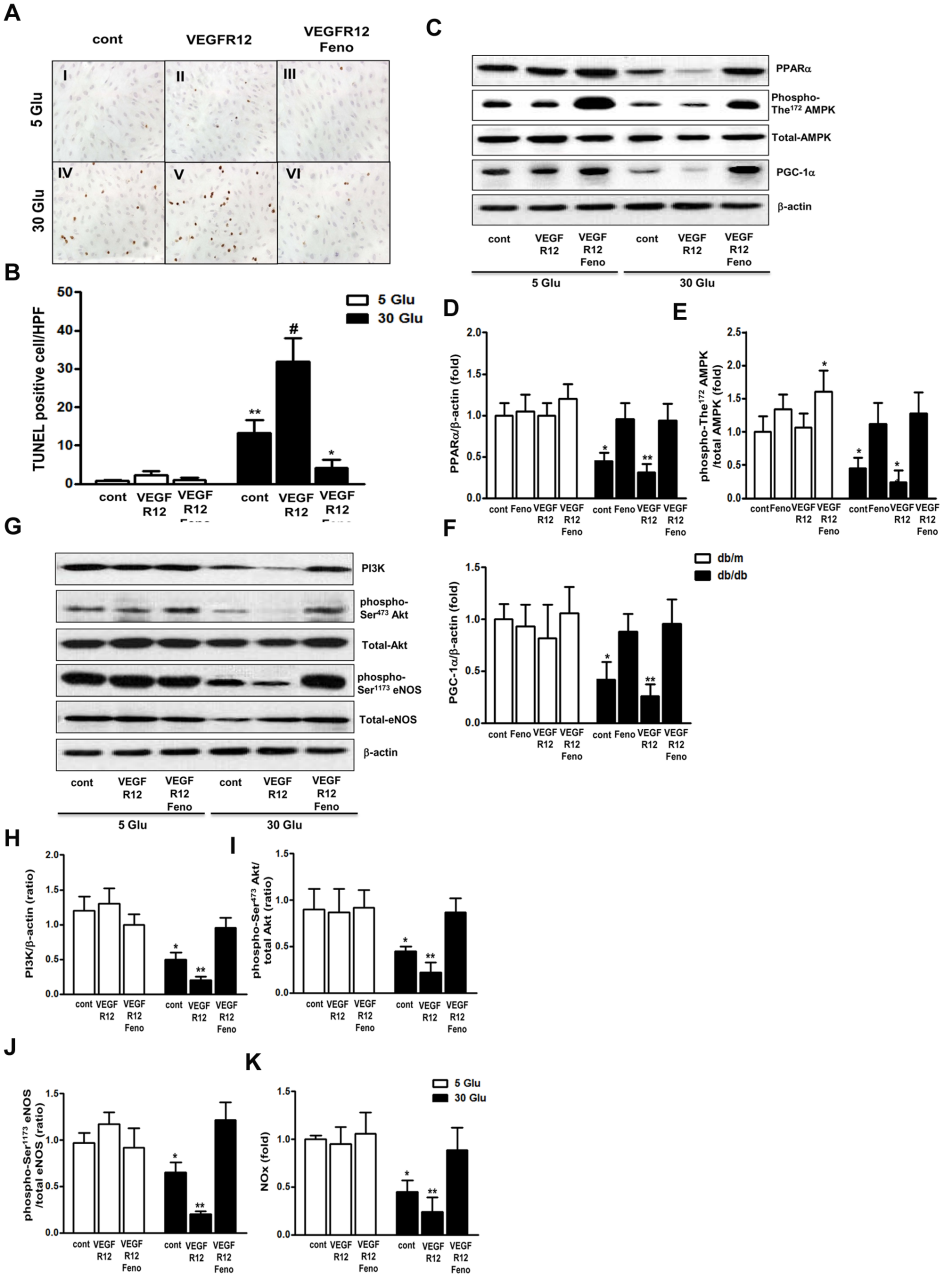
Fenofibrate prevents high glucose-induced oxidative stress and apoptosis in HUVECs under VEGF inhibition. Marked increases in TUNEL-positive human umbilical vein endothelial cells (HUVECs) were observed in the 30 Glu group and 30 Glu VEGFR12 compared to the 5 Glu group with or without fenofibrate treatment. Quantitative analyses of the results are shown (A and B). *P<0.05, **P<0.01 and ^#^ *P*<0.001 versus 5 Glu, 5 Glu VEGFR12, or 5 Glu VEGFR12+Feno groups. Analysis of intracellular PPARα-PGC-1α-phospho-The^172^/total AMPK pathway and PI3K-Akt-eNOS signaling and apoptosis in cultured HUVECs. Representative Western blot of PPARα, PGC-1α and phospho-The^172^/total AMPK, and β-actin and quantitative analyses of the results are shown (C to F). Representative Western blot of PI3K, total and phospho-Ser^473^ Akt, total and phospho-Ser^1173^ eNOS, and β-actin and quantitative analyses of the results are shown (G to J). The NOx concentrations from the supernatant of HUVECs are shown (K). *P<0.05 and **P<0.01 versus other groups. 5 Glu; 5 mmol/L D-glucose concentration, 5 Glu VEGFR12; 5 mmol/L D-glucose concentration with VEGFR12 inhibition, 5 Glu VEGFR12+Feno; 5 mmol/L D-glucose concentration with VEGFR12 inhibition+Feno; IV, 30 Glu; 30 mmol/L glucose, 30 Glu VEGFR12; 30 mmol/L D-glucose concentration with VEGFR12 inhibition; 30 Glu VEGFR12+Feno; 30 mmol/L D-glucose concentration with VEGFR12+Feno.

We next examined the effects of high glucose in combination with VEGFR inhibition and fenofibrate treatment, and found that the amount of TUNEL-positive HUVECs only increased in high-glucose medium (Figure 5; *P*<0.01). Furthermore, VEGFR inhibition augmented the increase in TUNEL-positive HUVECs in high-glucose medium. The number of TUNEL-positive cells in high-glucose medium was markedly decreased by fenofibrate. However, fenofibrate treatment did not cause any significant differences in the numbers of TUNEL-positive HUVECs in low-glucose and 25 mmol/L mannitol medium, either with or without VEGFR inhibition.

The intracellular PGC-1α-PI3K-Akt-eNOS signaling pathway is thought to play an important role in the maintenance of normal endothelial cell function [21], [22]. To examine the effects of VEGFR inhibition and fenofibrate treatment on endothelial cell apoptosis and dysfunction in HUVECs, we investigated the expression levels of PPARα, PGC-1α, phosphorylated AMPK and PI3K-Akt-eNOS signaling molecules in these cells. Significant decreases in the levels of phospho-AMPK(Thr^172^), a molecule involved in AMPK-PGC-1α signaling, phospho-Akt(Ser^473^), a molecule involved in PI3K-Akt signaling, and phospho-eNOS(Ser^1177^) were noted in high-glucose medium (Figure 5). The expression levels of these proteins were decreased to an even greater extent by VEGFR inhibition. Consistent with these intracellular signaling changes, extracellular NOx concentrations decreased in high-glucose medium and decreased to an even greater extent upon VEGFR inhibition (Figure 5). Such changes were not observed at low glucose levels or under osmotic control, suggesting that extracellular glucose suppresses PGC-1α-phospho-AMPK and PI3K-phospho-Atk-phospho-eNOS-NOx signaling pathways in HUVECs. Since fenofibrate stimulates PGC-α expression [25], [27], we also investigated the effects of VEGFR inhibition on PGC-1α expression levels and PGC-1α downstream signaling molecules in high-glucose medium. Notably, fenofibrate enhanced PGC-1α expression and AMPK phosphorylation. Moreover, fenofibrate activated PI3K-Akt and eNOS-NOx signaling pathways to levels similar to those observed in low-glucose medium.

**Figure 6:**
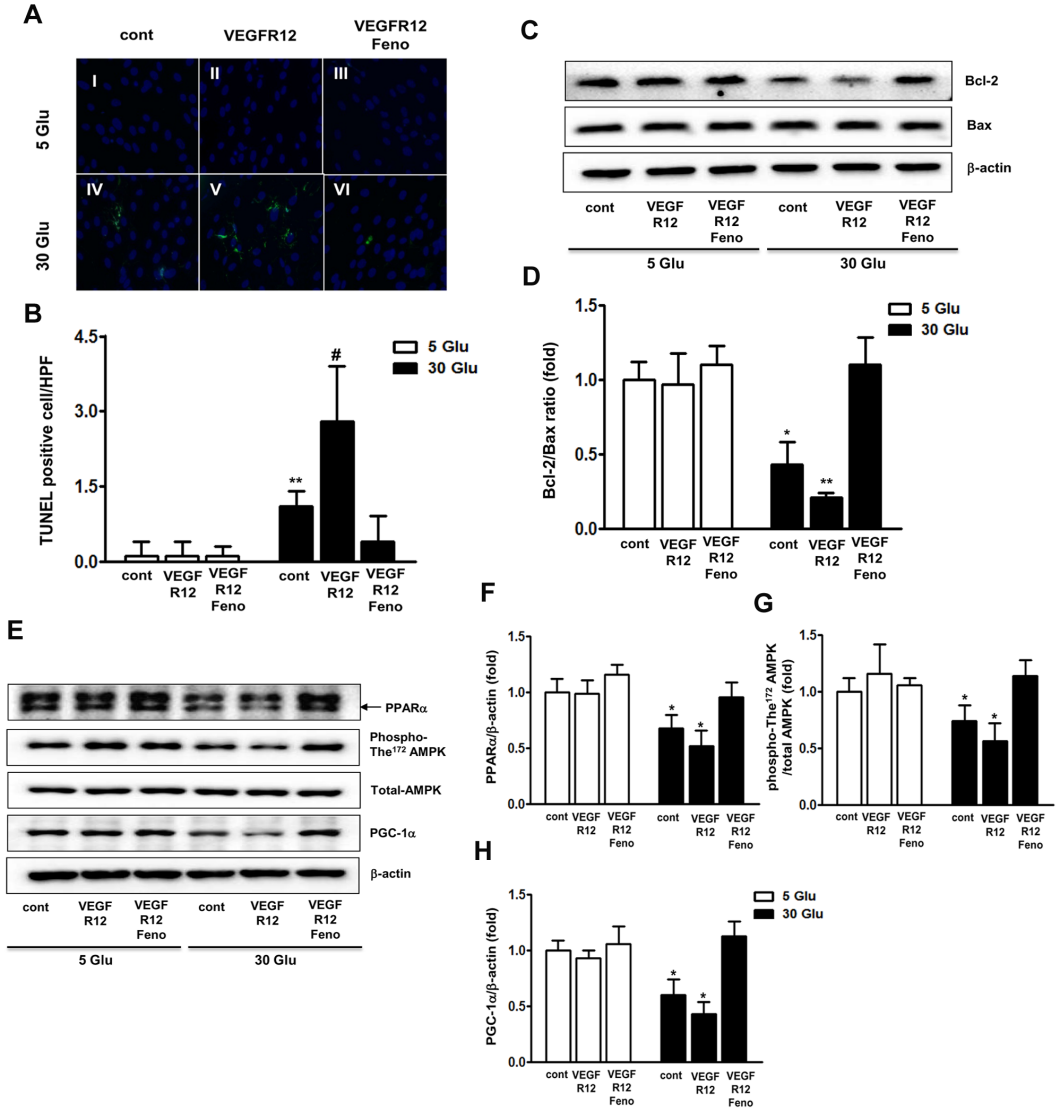
Fenofibrate prevents high glucose-induced oxidative stress and apoptosis in HSCs under VEGF inhibition. Marked increases in TUNEL-positive human Schwann cells (HSCs) were observed in the 30 Glu group and 30 Glu VEGFR12 compared to the 5 Glu group with or without fenofibrate treatment. Quantitative analyses of the results are shown (A and B, original magnification, ×1,000). **P<0.01 and ^#^ *P*<0.001 versus 5 Glu, 5 Glu VEGFR12, or 5 Glu VEGFR12+Feno groups. Western blot analysis of Bcl-2, Bax, and β-actin and quantitative analyses of the results are shown (C and D). *P<0.05 and **P<0.01 versus *db/m* controls. Analysis of intracellular PPARα-phospho-The^172^/total AMPK-PGC-1α pathway in cultured HSCs. Representative Western blot of PPARα, phospho-The^172^/total AMPK, PGC-1α, and β-actin and quantitative analyses of the results are shown (E to H). *P<0.05 and **P<0.01 versus other groups. 5 Glu; 5 mM D-glucose concentration, 5 Glu VEGFR12; 5 mM D-glucose concentration with VEGFR12 inhibition, 5 Glu VEGFR12+Feno; 5 mM D-glucose concentration with VEGFR12 inhibition+Feno; IV, 30 Glu; 30 mM glucose, 30 Glu VEGFR12; 30 mM D-glucose concentration with VEGFR12 inhibition; 30 Glu VEGFR12+Feno; 30 mM D-glucose concentration with VEGFR12+Feno.

We also examined the effects of a high-glucose environment, either alone or in combination with VEGFR inhibition and fenofibrate treatment, on HSCs. Initially, we observed a significant increase in the number of TUNEL-positive HSCs when cells were cultured in high-glucose medium alone (Figure 6). Upon VEGFR inhibition in high-glucose medium, the number of TUNEL-positive HSCs was increased to an even greater extent. However, fenofibrate treatment markedly decreased the number of TUNEL-positive cells, even when cells were cultured in high-glucose medium. Furthermore, this decrease was also associated with a change in the Bcl-2/Bax ratio (Figure 6). However, no significant difference in the number of TUNEL-positive HUVECs was found when cells grown in low-glucose medium supplemented with 25 mmol/L mannitol (either with or without VEGFR inhibition) were treated with fenofibrate. The decrease in PPARα-AMPK-PGC-1α signaling observed in HSCs grown in high-glucose medium was markedly increased by fenofibrate treatment (Figure 6), suggesting that fenofibrate exerts a protective effect on the sciatic nerve and prevents the vascular damage inflicted by additional VEGFR12 treatment in *db/db* animals. In vitro studies also revealed that VEGFR12 treatment decreased the expression levels of key proteins in the PPARα-AMPK-PGC-1α signaling pathway, indicating that the effects of VEGFR12 on endothelial cell and neural damage, especially in Schwann cells, and the effects of fenofibrate on rescuing this damage, are mediated at least in part through the same pathway.

## Discussion

The results presented here demonstrate that inhibition of VEGFRs with anti-VEGFR1 (GNQWFI) and anti-VEGFR2 (ATWLPPR) peptides aggravates DPN in type 2 diabetic mice. Inhibition of VEGFRs led to a decrease in the number of endoneurial blood vessels, a decrease in the area of unmyelinated fiber, a decrease in axonal diameter, and an increase in the amount of axonal degeneration in the sciatic nerve. These effects are all characteristics of human DPN, indicating that hypoxic nerve injury results from endoneurial and endothelial cell damage. In contrast, fenofibrate treatment largely prevented the development of DPN, in a manner independent of VEGFRs, by activating the PPARα-AMPK-PGC-1α pathway. Moreover, in vitro studies provided direct evidence for the preventative effects of fenofibrate against high glucose-induced endothelial cell damage. In these studies, fenofibrate activated the PPARα-AMPK-PGC-1α signaling pathway, leading to subsequent activation of downstream molecules such as PI3K and those involved in Akt-eNOS-NOx signaling. These findings are consistent with the previously proposed role of PPARα in PGC-1α activation, including the subsequent activation of the PI3K-Akt-eNOS signaling pathway [23], [24]. Through this pathway, PPARα attenuates vascular damage in various ways, including reducing lipotoxicity, inflammation, the generation of reactive oxygen species, endothelial dysfunction, and angiogenesis in DPN. In addition, studies using HSCs cultured in vitro demonstrated that VEGFR inhibition decreased the expression levels of key proteins involved in PPARα-AMPK-PGC-1α signaling, indicating that the mechanisms by which VEGFR inhibition and hyperglycemia mediate nerve damage, and by which fenofibrate exerts a protective effect against this damage, require at least in part the same pathway.

The pathogenesis of DPN is not yet fully understood; however, ischemia and oxidative stress are thought to be involved [4], [16], [17]. The development of neuropathy in DPN may depend on the severity of hyperglycemia, which gradually damages the endothelium [33], [34]. Serum lipid abnormalities, particularly elevated TG levels, appear to be strong predictors of microvascular complications in the neuron [35]. In the present study, no differences in TG levels were found in *db/db* mice, either with or without fenofibrate treatment. It is possible that fenofibrate-induced concomitant increases in HDL-C levels and decreases in blood glucose concentrations may have protective effects against DPN.

Microvascular insufficiency-related ischemia causes neuronal dysfunction, even in the absence of other metabolic abnormalities, because neuropathological changes in the peripheral nerves of non-diabetic patients with chronic hypoxia are quite similar to those found in DPN patients [4]. Vascular changes may be associated with secondary deficits in endothelial function resulting from impaired blood flow, such as impaired NO synthesis, release of NO upon endothelial injury by oxidative stress, and increased free radical activity [36]. Our study clearly demonstrates that VEGFR inhibition in diabetic mice increased endothelial and nerve damage, as indicated by a decrease in the number of PECAM-1-positive cells in the endoneurial blood vessels and increases in the numbers of TUNEL-positive, F4/80-positive, and PECAM-1 and TUNEL-double positive cells in the sciatic nerve upon VEGFR inhibition.

PPARα activation is known to have tissue-specific effects. In the nervous system, activated PPARα appears to be involved in neuronal differentiation, inflammation, and autoimmune modulation, and also protects nerves against ischemia [37], [38]. Studies of retinal endothelial cells and HUVECs have demonstrated that fenofibrate- or pirinixic acid- (a PPARα agonist) mediated activation of AMPK increases NO production, inhibits NF-κB, and suppresses expression of genes encoding adhesion molecules [25], [39], [40]. These results suggest that endothelial cell protection and metabolic activities are tightly coordinated; however, it had not previously been determined whether diabetes affects PPARα or PGC-1α expression. Interestingly, PGC-1α is a metabolic sensor whose role is to stimulate angiogenesis in ischemic tissue such as in the nerve [19]. In the present study, diabetes decreased PGC-1α expression levels and suppressed AMPK-Akt-eNOS signaling. In the sciatic nerve, reduction of PPARα expression levels was especially pronounced upon VEGF inhibition; however, fenofibrate treatment restored PPARα expression to non-diabetic levels. These findings are consistent with a previous study demonstrating that fenofibrate suppressed microvascular inflammation and apoptosis by activating AMPK-Akt-eNOS signaling [41]. Various cytoprotective mechanisms of PGC-1α have been postulated, including activation of PI3K/Akt and secondary attenuation of p38 signaling, increased expression of survivin, and upregulation of Bcl-2 and/or inhibition of caspase-3 activity [41], [42], [43]. Most of these cytoprotective functions are associated with VEGF activation. In contrast, a recent study found that exercise-induced PGC-1α activation in the skeletal muscle of untrained elderly subjects actually increased angiogenesis, without activating VEGF [44]. In our study, we found that fenofibrate protected against endothelial and Schwann cell damage, and that the mechanism of fenofibrate-mediated protection of the sciatic nerve involves activation of the AMPK-PGC-1α signaling pathway by PPARα, in a manner independent of VEGF.

In conclusion, this study demonstrates that hyperglycemia in diabetic mice aggravates DPN by increasing endothelial cell damage and inflammation of the sciatic nerve, and that these effects are aggravated by VEGF inhibition. Fenofibrate treatment prevented endothelial cell damage and inflammation of the sciatic nerve by activating PPARα-AMPK-PGC-1α signaling, in a VEGF-independent manner, leading to subsequent activation of PI3K-Akt-eNOS signaling in the sciatic nerve, HUVECs, and HSCs. Thus, our results provide evidence that fenofibrate exerts neuroprotective actions, such as ameliorating endothelial and/or nerve cell damage, at least in Schwann cells, and dampening inappropriate inflammation responses.
